# Neuromuscular degeneration and locomotor deficit in a *Drosophila* model of mucopolysaccharidosis VII is attenuated by treatment with resveratrol

**DOI:** 10.1242/dmm.036954

**Published:** 2018-11-20

**Authors:** Sudipta Bar, Mohit Prasad, Rupak Datta

**Affiliations:** Department of Biological Sciences, Indian Institute of Science Education and Research (IISER) Kolkata, Mohanpur 741246, West Bengal, India

**Keywords:** β-glucuronidase, Mucopolysaccharidosis, Neuromuscular degeneration, Resveratrol

## Abstract

Mucopolysaccharidosis VII (MPS VII) is a recessively inherited lysosomal storage disorder caused by β-glucuronidase enzyme deficiency. The disease is characterized by widespread accumulation of non-degraded or partially degraded glycosaminoglycans, leading to cellular and multiple tissue dysfunctions. The patients exhibit diverse clinical symptoms, and eventually succumb to premature death. The only possible remedy is the recently approved enzyme replacement therapy, which is an expensive, invasive and lifelong treatment procedure. Small-molecule therapeutics for MPS VII have so far remained elusive primarily due to lack of molecular insights into the disease pathogenesis and unavailability of a suitable animal model that can be used for rapid drug screening. To address these issues, we developed a *Drosophila* model of MPS VII by knocking out the *CG2135* gene, the fly β-glucuronidase orthologue. The *CG2135*^−/−^ fly recapitulated cardinal features of MPS VII, such as reduced lifespan, progressive motor impairment and neuropathological abnormalities. Loss of dopaminergic neurons and muscle degeneration due to extensive apoptosis was implicated as the basis of locomotor deficit in this fly. Such hitherto unknown mechanistic links have considerably advanced our understanding of the MPS VII pathophysiology and warrant leveraging this genetically tractable model for deeper enquiry about the disease progression. We were also prompted to test whether phenotypic abnormalities in the *CG2135*^−/−^ fly can be attenuated by resveratrol, a natural polyphenol with potential health benefits. Indeed, resveratrol treatment significantly ameliorated neuromuscular pathology and restored normal motor function in the *CG2135*^−/−^ fly. This intriguing finding merits further preclinical studies for developing an alternative therapy for MPS VII.

This article has an associated First Person interview with the first author of the paper.

## Abstract (precis)

**Summary:** The authors generate a new *Drosophila* model of MPS VII, which offers insights into the pathophysiology of this disease, and also uncovers the therapeutic potential of resveratrol in treating MPS VII.

## INTRODUCTION

Mucopolysaccharidosis VII (MPS VII), also known as Sly syndrome, is a recessively inherited lysosomal storage disorder caused by β-glucuronidase (β-GUS) deficiency. Because of heterogeneity of mutations in the *β-GUS* gene (*GUSB*) that results in varying extents of enzyme deficiency, MPS VII patients exhibit diverse clinical symptoms, ultimately leading to premature death in most cases. Short stature, cognitive disability, skeletal abnormality, motor impairment, hernias, hepatosplenomegaly, hydrops fetalis, and heart and respiratory problems are some of the common clinical signs seen in MPS VII. Many of these symptoms are also manifested in other MPS disorders, suggesting a common pathophysiological mechanism for this group of disorders (Montaño et al., 2016; Neufeld and Muenzer, 2014; Sly et al., 1973; Zielonka et al., 2017). Until recently, there was no treatment for MPS VII. However, a clinical trial for enzyme replacement therapy with recombinant human β-GUS led to FDA approval of the drug in November, 2017 (Fox et al., 2015; Harmatz et al., 2018).

β-GUS is one of the eleven lysosomal hydrolases responsible for stepwise degradation of complex polysaccharides, glycosaminoglycans (GAGs) (Neufeld and Muenzer, 2014). Loss of its activity leads to accumulation of engorged lysosomes, containing non-degraded or partially degraded GAGs, in various cells and tissues of the affected individuals (Irani et al., 1983; Vogler et al., 1994). A fraction of the non-degraded GAGs are also secreted into the blood stream and excessive amounts are excreted in urine (Sewell et al., 1982). Although ∼50 disease-causing mutations in the *β-GUS* gene have been reported to date, the molecular events that lead from enzyme deficiency and GAG storage to multiple tissue dysfunction or damage is poorly understood (Tomatsu et al., 2009).

In 1989, Birkenmeier et al. characterized a natural mutant mouse lacking β-GUS activity that recapitulates many features of MPS VII patients. The mutant male mice were reproductively sterile. The mutant females were fertile but they suffered from insufficient lactation and hence could not nurture their pups (Birkenmeier et al., 1989). Even heterozygous mating produced a less-than-expected number of pups because of neonatal death, thus making it extremely difficult to produce these animals in large quantities (Soper et al., 1999). Despite these challenges, this mutant mouse, which was later found to carry a frameshift mutation in the *β-GUS* gene, became the mainstay of MPS VII research (Sands and Birkenmeier, 1993). Among all the affected tissues, brain pathology is relatively well studied in the MPS VII mouse model (Heuer et al., 2002; Levy et al., 1996). Although vacuolar storage lesions were uniformly distributed throughout the brain, signs of neurodegeneration were only seen in discrete regions, particularly in the hippocampus and cerebral cortex (Heuer et al., 2002). Upregulation of several inflammatory genes was observed in the posterior cortex of the diseased brain (Richard et al., 2008). Many pro-apoptotic genes were also found to be transcriptionally activated in the MPS VII mouse brain but, surprisingly, no apoptotic cells could be detected in those tissues (Heuer et al., 2002; Richard et al., 2008). However, the molecular basis of selective neurodegeneration in MPS VII is still unclear. Also, how lysosomal storage affects non-neuronal tissues remains to be investigated.

In recent times, many neurodegenerative diseases, including some of the lysosomal storage disorders like Niemann-Pick disease, mucolipidosis type IV and Gaucher disease, have been successfully modelled in *Drosophila* (Bonini and Fortini, 2003; Feany and Bender, 2000; Huang, 2005; Iijima et al., 2004; Kinghorn et al., 2016; Venkatachalam et al., 2008). These simple, genetically tractable fly models have led to better understanding of the mechanism of these diseases and also have provided platforms for translational research. Encouraged by these studies, we decided to develop a *Drosophila* model of MPS VII. We first identified the functional *β-GUS* orthologue in *Drosophila* (annotated as *CG2135* in the FlyBase; also known as *betaGlu*) and then generated a *CG2135*^−/−^ knockout fly. The *CG2135*^−/−^ flies were viable with no overt phenotype. Crosses between them produced the expected number of embryos but they had a somewhat reduced hatching rate, possibly a consequence of a low-penetrance developmental defect. The adult *CG2135*^−/−^ fly exhibited typical features of MPS VII, including a reduced lifespan, progressive decline in locomotor activity and abnormal neuropathology. This fly also had increased accumulation of storage materials in the brain, which is the pathological hallmarks of lysosomal storage disorders. Neurodegeneration, especially the loss of dopaminergic neurons, and extensive muscle atrophy was implicated as the basis of locomotor disability in this fly. Interestingly, the neuromuscular pathology as well as the locomotor defect in the *CG2135*^−/−^ fly was restored to a significant extent upon treatment with resveratrol, a natural polyphenol (Baur and Sinclair, 2006). Therefore, in addition to providing mechanistic insights into the pathogenesis of MPS VII, our work uncovered a therapeutic lead for managing this debilitating disease. This novel fly model may have a far-reaching impact as it opens up the opportunity for rapid screening of potential drugs that would not be feasible with the existing MPS VII mouse (Fernández-Hernández et al., 2016). It also offers an excellent system for conducting genetic screens to gain deeper understanding of the disease progression (St Johnston, 2002).

## RESULTS

### Identification of *CG2135* as an active *β-GUS* orthologue in *Drosophila*

**Fig. 1.**
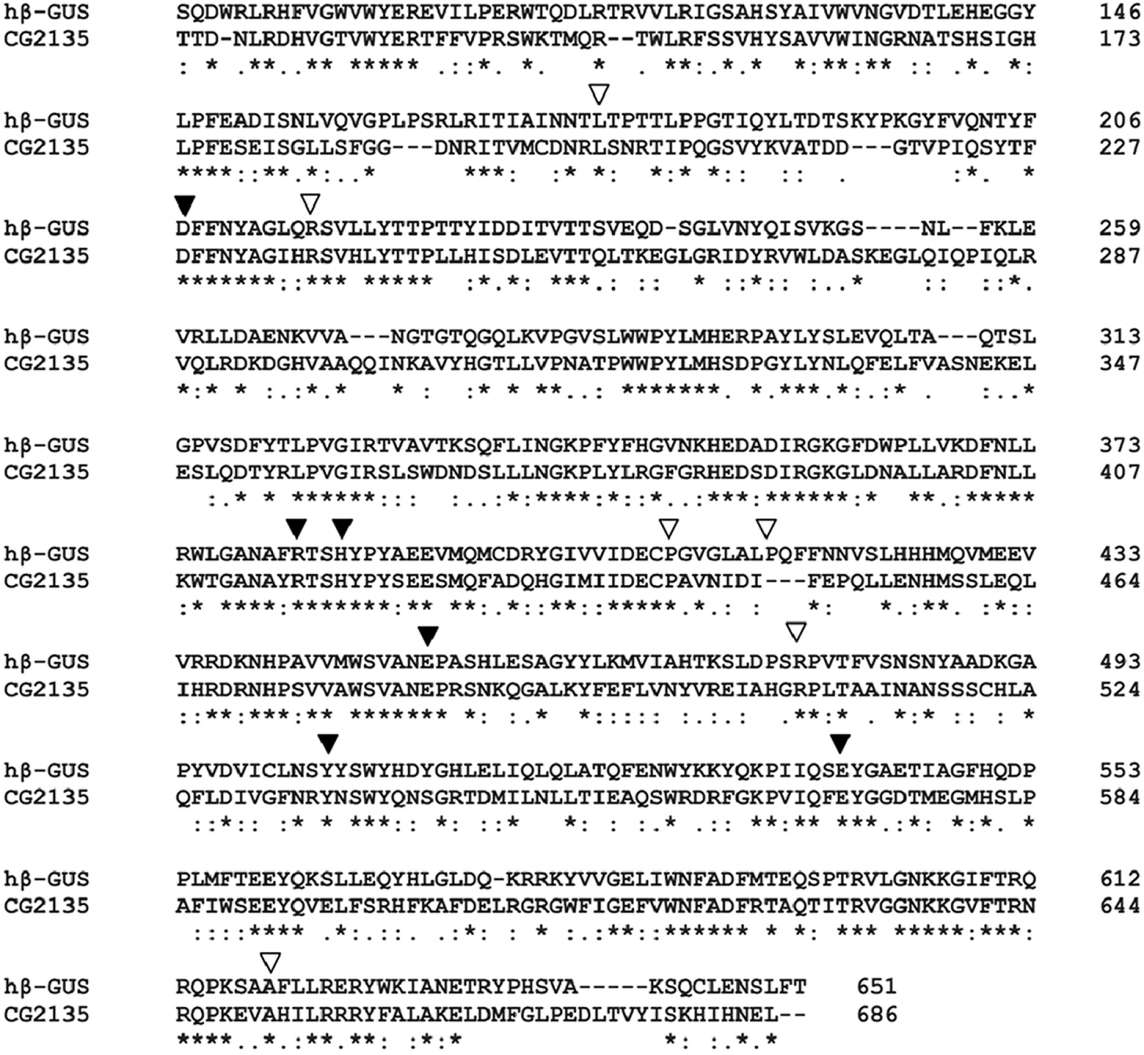
**Sequence alignment of human and *Drosophila* β-GUS.** Amino acid sequence alignment of *Drosophila* β-GUS (annotated as CG2135 in FlyBase) and human β-GUS (hβ-GUS; UniProt accession number P08236). The active-site residues of hβ-GUS and most frequently mutated residues in MPS VII are marked by black and white triangles, respectively. An asterisk (*) indicates positions with a single, fully conserved residue; a colon (:) indicates conservation between groups of strongly similar properties; and a period (.) indicates conservation between groups of weakly similar properties.

We first confirmed that an enzymatically active β-GUS is ubiquitously expressed in all developmental stages of *Drosophila* and in all tissues of the adult fly that were analyzed (Table 1). Simultaneously, we identified a putative β-GUS-encoding gene in the *Drosophila* genome, annotated as *CG2135* (http://flybase.org). The CG2135 protein was predicted to possess an N-terminal endoplasmic reticulum (ER)-targeting signal peptide, which is characteristic of all lysosomal hydrolases (Fig. S1) (Kornfeld, 1987). Sequence alignment revealed that CG2135 shares >40% identity and >60% similarity with that of the human β-GUS protein. Critical amino acids of the human β-GUS active site as well as most frequently mutated residues in MPS VII were conserved in CG2135 (Fig. 1) (Hassan et al., 2013). Widespread expression of *CG2135* mRNA was confirmed by reverse transcription (RT)-PCR, which is consistent with a previous microarray-based transcriptome analysis (Fig. S2) (Chintapalli et al., 2007). Next, *CG2135* cDNA was cloned, the protein expressed in S2 cells, purified by Ni-affinity chromatography and finally subjected to β-GUS assay. The authenticity of CG2135 as a functional β-GUS was confirmed by its specific activity of 3.7×10^6^ units/mg, which is comparable to the previously reported activity of human β-GUS. Similar to human β-GUS, CG2135 was found to be a thermally stable enzyme, active over a wide range of pH and with an optimum pH∼5.0 (Fig. S3A-C) (Grubb et al., 2008; Islam et al., 1999).

**Table 1.**
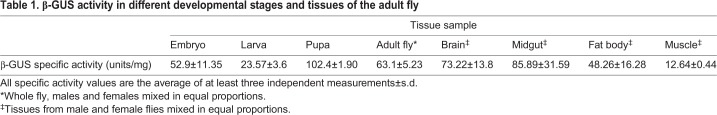
**β-GUS activity in different developmental stages and tissues of the adult fly**

### Generation of the *CG2135*^−/−^ fly by targeted gene disruption

**Fig. 2.**
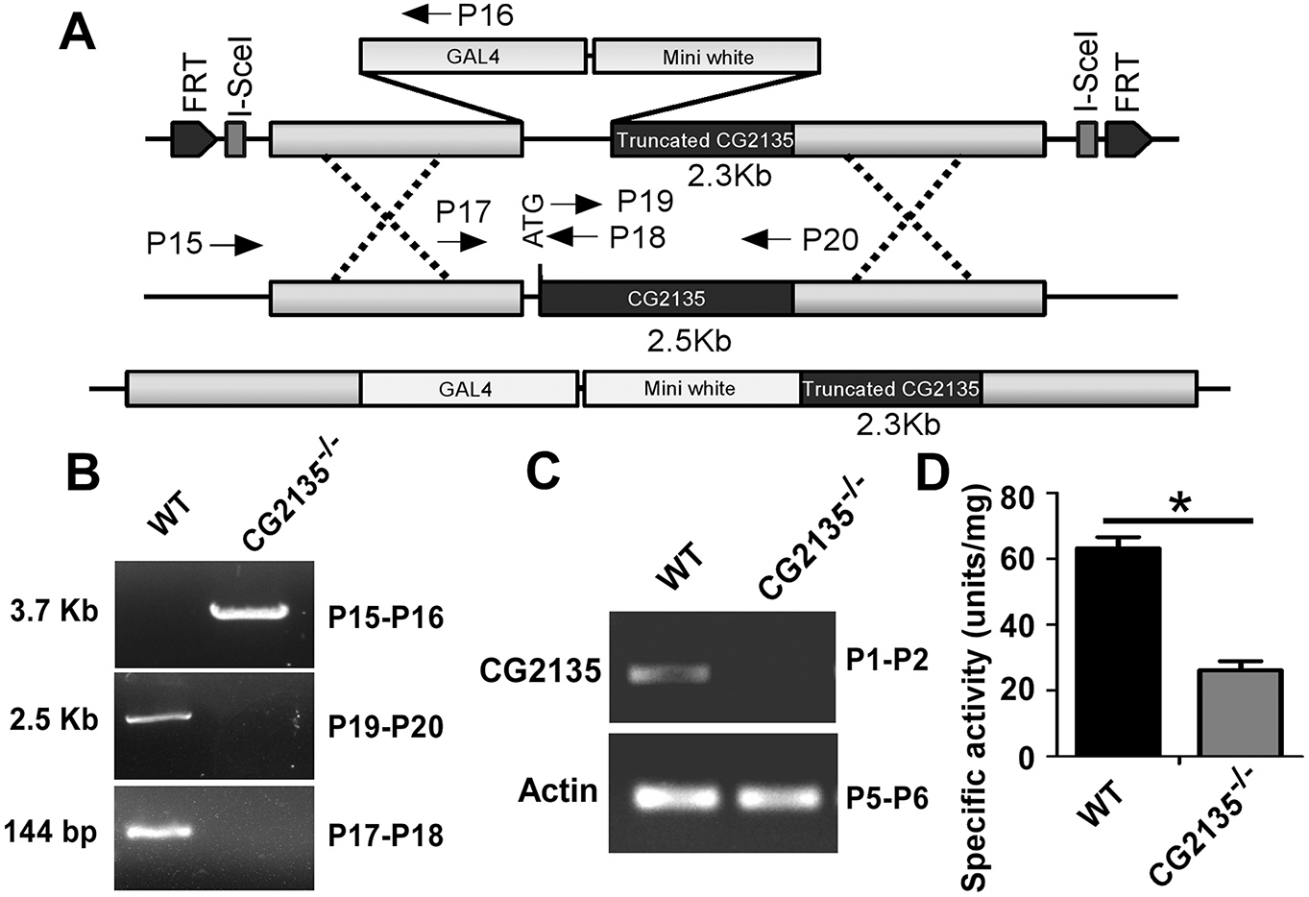
**Generation of the *CG2135*^−/−^ fly.** (A) Schematic representation of the linearized *CG2135* knockout construct containing *Gal4* and mini-*white* markers flanked by 5′ and 3′ genomic regions of *CG2135* with a truncation of a 212-bp fragment. FRT and I-*Sce*I sites are also shown. The targeted allele after homologous recombination is shown at the bottom. Primer binding sites are indicated by arrows. (B) Genotype of the wild-type (WT) and the *CG2135*^−/−^ fly was verified by genomic PCR. Integration of the *Gal4* marker gene in the *CG2135*^−/−^ fly was confirmed by PCR amplification of the 3.7-kb product with P15-P16 primers. Deletion of the genomic fragment in the *CG2135*^−/−^ fly was verified by the absence of any PCR products with P19-P20 and P17-P18 primer sets. The 2.5-kb and 144-bp PCR products were amplified in the WT fly, as expected. (C) RT-PCR with *CG2135*-specific primer sets (P1-P2), showing absence of the *CG2135* mRNA in the *CG2135*^−/−^ fly. RT-PCR with actin amplification (P5-P6) served as control. (D) β-GUS-specific activity in the WT and *CG2135*^−/−^ fly. Error bars represent s.e.m. of values from three independent experiments. *P≤0.05.

Having confirmed *CG2135* as an authentic *β-GUS* orthologue, our next goal was to generate a *CG2135*^−/−^ fly. For this, a targeting construct was generated consisting of the *Gal4* and mini-*white* markers flanked by 5′ and 3′ genomic regions of *CG2135*. Homologous recombination between this targeting construct and the endogenous locus produced the targeted allele, in which a 212-bp fragment of the *CG2135* coding sequence (including the start codon) was replaced with the *Gal4* and mini-*white* markers, thus resulting in a genetic deletion as well as a frameshift (Fig. 2A). The targeting event in the *CG2135*^−/−^ fly was verified by genomic PCR with primers (P15-P16) that bind to the genomic sequence outside the targeted region and to the *Gal4* gene. No PCR product was amplified from genomic DNA of the wild-type fly. Additionally, genomic PCR was performed with primers (P17-P18 and P19-P20) that bind inside the targeted region and to the region which gets deleted after recombination. PCR amplification was observed only with genomic DNA from the wild-type fly but not from the *CG2135*^−/−^ fly, which harbours the deletion (Fig. 2A,B). Knockout of the *CG2135* gene was further confirmed by the complete absence of the *CG2135* mRNA in the *CG2135*^−/−^ fly (Fig. 2C). Interestingly, β-GUS activity in the *CG2135*^−/−^ fly, although reduced to a significant extent, was not totally abolished (Fig. 2D). To check whether this residual β-GUS activity in the *CG2135*^−/−^ fly is contributed by another β-GUS-like protein, we performed a blast search of the *Drosophila* genome with the CG2135 protein sequence. This revealed existence of a gene of unassigned function (*CG15117*) that has high amino-acid sequence similarity with that of the *CG2135*. This finding is consistent with an older report, where two chromatographically separable forms of β-GUS were shown to exist in *Drosophila* (Langley et al., 1983). Unlike lysosomal hydrolases, CG15117 lacked the N-terminal ER-targeting signal peptide, suggesting it to be a non-lysosomal enzyme that plays some non-conventional role (Fig. S4A,B). Purified CG15117 was found to be six-fold less active than CG2135 (Fig. S5A,B). The *CG15117* mRNA was expressed in the wild-type as well as in the *CG2135*^−/−^ fly, thereby explaining why a low level of β-GUS activity could be detected in tissues after complete abolition of *CG2135* expression (Fig. S5C).

### Shortened lifespan and progressive decline of locomotor activity in the *CG2135*^−/−^ fly

**Fig. 3.**
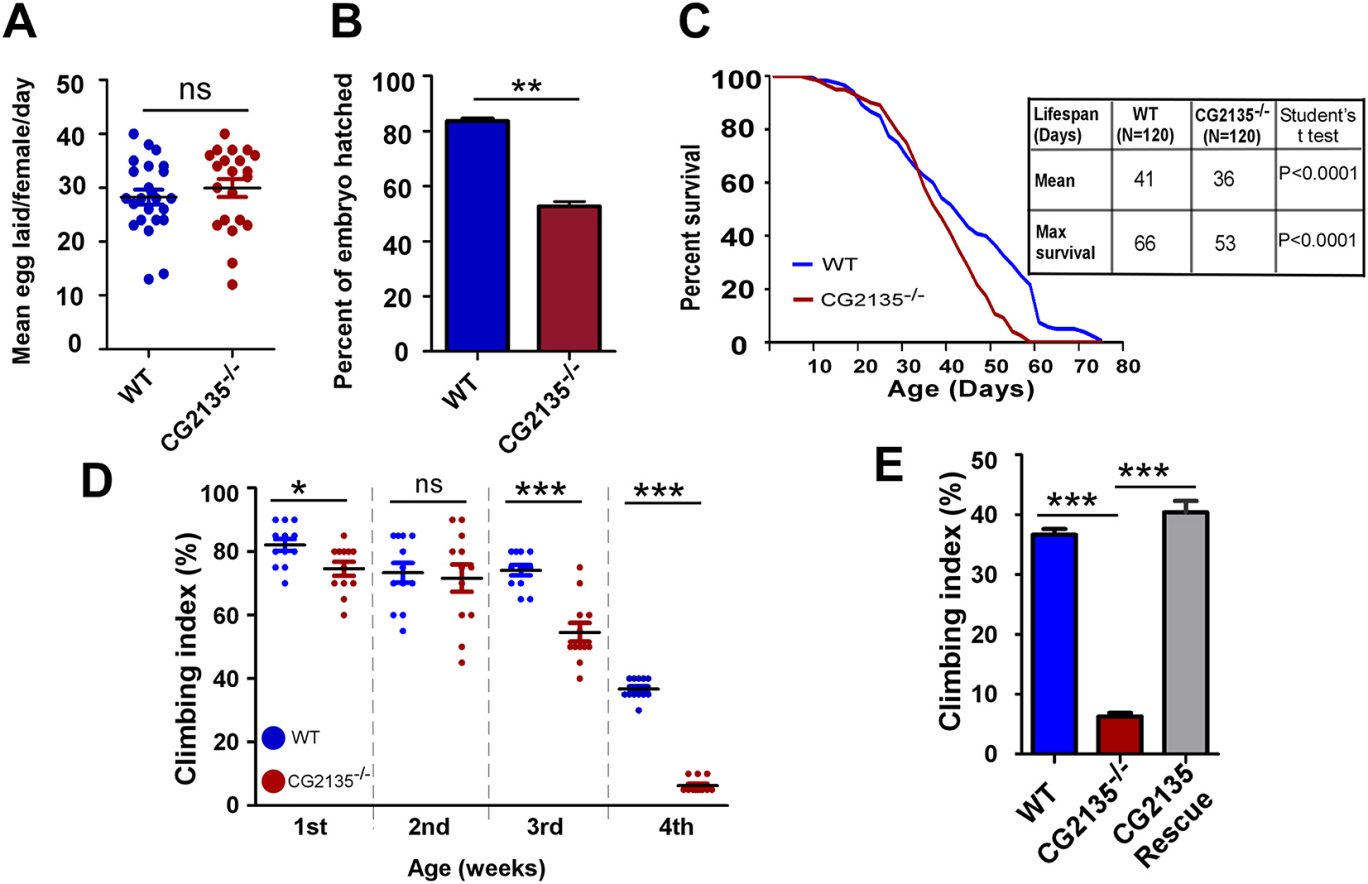
**Phenotypic abnormalities in the *CG2135*^−/−^ fly.** (A) Scatter plot showing the wild-type (WT) and the *CG2135*^−/−^ fly both produce a similar number of embryos per day. Line represents the mean value; error bar represents s.e.m. and dots represent individual data point (*N*>20). (B) Bar graph showing a significantly reduced hatching percentage of the *CG2135*^−/−^ embryos compared to the WT (*N*=150). (C) Survival curve of the WT and the *CG2135*^−/−^ flies. Mean and maximum lifespan values are provided in the adjacent table. (D) Scatter plot representing climbing index of WT and *CG2135*^−/−^ flies of different ages (*N*∼200). (E) Bar graph showing a significant decline in climbing index of the 4-week-old *CG2135*^−/−^ flies as compared to the age-matched WT flies. Complete restoration of climbing activity was observed in the age-matched rescue fly (*N*∼200). Error bar represents s.e.m. of values from at least three independent experiments. ‘ns’ indicates not significant. *P≤0.05, **P≤0.01, ***P≤0.001.

The *CG2135*^−/−^ homozygous flies were viable and did not show any gross abnormalities. Crosses between them produced the expected number of embryos but they had ∼30% less than normal hatching rate, suggesting that loss of *CG2135* resulted in an early developmental defect with incomplete penetrance (Fig. 3A,B). Compared to the wild-type flies, adult *CG2135*^−/−^ flies had reduced survivability as indicated by their shortened mean and maximum lifespan by 5 and 13 days, respectively (Fig. 3C). The *CG2135*^−/−^ flies also exhibited age-dependent impairment of locomotor activity as analyzed by negative geotaxis (climbing) assay (Strauss and Heisenberg, 1993; Venkatachalam et al., 2008). While the adult *CG2135*^−/−^ flies had almost no climbing disability until the second week, their performance worsened from the third week onwards. At the fourth week, they exhibited ∼85% decline in climbing ability compared to their wild-type counterparts (Fig. 3D). To confirm that the observed locomotor defect is a direct consequence of loss of CG2135 function, we created a transgenic fly overexpressing *CG2135* on the *CG2135*^−/−^ background (Fig. S6). Complete restoration of locomotor function was observed in this fly (Fig. 3E). The features of the *CG2135*^−/−^ flies reported here are thus reminiscent of the movement disability and reduced life expectancy seen in MPS VII patients (Montaño et al., 2016).

### Neuropathological abnormalities in the *CG2135*^−/−^ fly

**Fig. 4.**
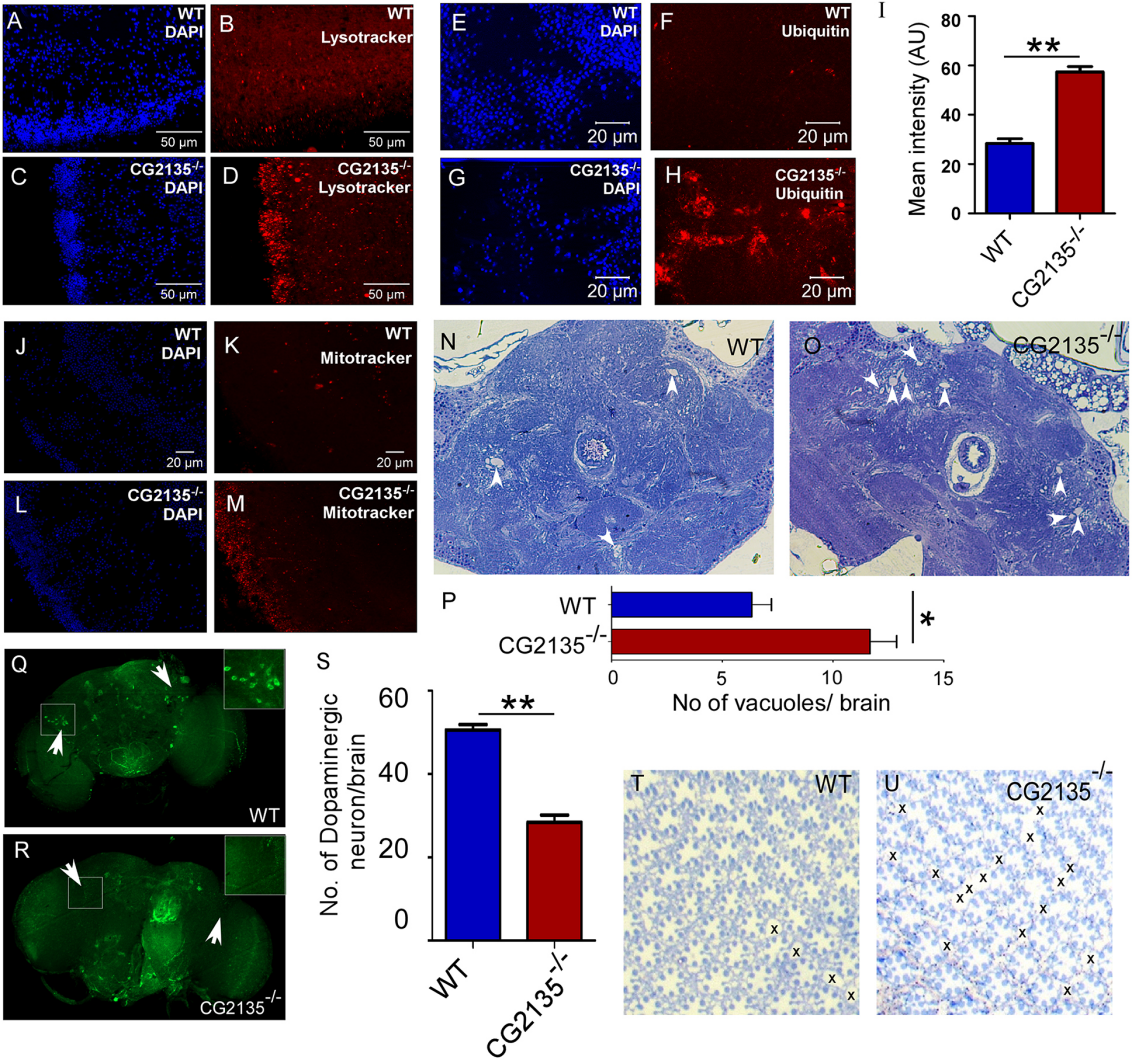
**Neuropathological abnormalities in the *CG2135*^−/−^ fly.** (A-D) LysoTracker (red) and DAPI (blue) staining of 30-day-old fly brains, showing increased abundance of the LysoTracker-positive vesicles in the *CG2135*^−/−^ fly compared to the wild type (WT). (E-H) Immunostaining of the 30-day-old fly brains with anti-ubiquitin antibody (red), showing an elevated level of ubiquitinated proteins in the *CG2135*^−/−^ fly. (I) Bar graph showing a significant increase in mean intensity of ubiquitin staining in the *CG2135*^−/−^ fly brain. (J-M) MitoTracker (red) and DAPI (blue) staining of the 30-day-old fly brains, showing increased mitochondrial accumulation in the *CG2135*^−/−^ fly brain. (N,O) Toluidine-Blue-stained brain sections of the 30-day-old flies, showing increased vacuolation (indicated by arrowheads) in the *CG2135*^−/−^ fly. (P) The number of vacuoles in the WT and *CG2135*^−/−^ fly brain sections. (Q,R) Immunostaining of the 30-day-old fly brains with anti-tyrosine-hydroxylase antibody (green), showing loss of dopaminergic neurons in the *CG2135*^−/−^ fly brain compared to the WT flies. Enlarged view of a dopaminergic neuron cell body from the boxed regions of the brain is provided in the inset. (S) Quantitation of dopaminergic neurons in the WT and *CG2135*^−/−^ fly brains. (T,U) Toluidine-Blue-stained retinal sections showing well-preserved ommatidial architectures in the 30-day-old WT fly, as opposed to defective ommatidial organization (‘x’ showing the gaps) in the age-matched *CG2135*^−/−^ fly. Error bar represents s.e.m. of values from at least three independent experiments. *P≤0.05, **P≤0.01.

Since locomotor activities are primarily controlled by brain and muscle function, we analyzed brain pathologies in *CG2135*^−/−^ flies to understand the underlying cause of their climbing defect (Flash and Hochner, 2005). First, 30-day-old fly brains were stained with LysoTracker Red to detect acidic organelles such as lysosomes and autolysosomes (Chazotte, 2011). Increased abundance of LysoTracker-positive vesicles was observed in the *CG2135*^−/−^ fly brain, which is consistent with accumulation of engorged lysosomes seen in patients suffering from MPS VII and other lysosomal storage disorders (Fig. 4A-D) (Ferreira and Gahl, 2017; Irani et al., 1983; Vogler et al., 1994). As compared to the age-matched controls, the *CG2135*^−/−^ flies exhibited elevated levels of ubiquitinated proteins in their brain (Fig. 4E-I). Interestingly, we also observed a significant increase in mitochondrial population in the *CG2135*^−/−^ fly brain (Fig. 4J-M). This abnormal build-up of ubiquitinated proteins and mitochondria might be due to a defect in the lysosome-mediated cellular clearance machinery that has been reported in many lysosomal storage disorders and other neurodegenerative diseases (Heuer et al., 2002; Osellame and Duchen, 2014; Platt et al., 2012). Examination of Toluidine-Blue-stained brain sections revealed increased vacuolation in the 30-day-old *CG2135*^−/−^ flies with respect to the age-matched controls (Fig. 4N-P). Those vacuolar lesions were mostly seen in the central complex region, which is associated with locomotor function in flies (Fig. 4O) (Strauss, 2002). The brain dopaminergic system is a critical modulator of locomotion in flies as well as in mammals (Riemensperger et al., 2013; Zhou and Palmiter, 1995). Also, movement disorders such as Parkinson's disease and progressive supranuclear palsy are characterized by loss of the dopaminergic neurons (Auluck et al., 2002; Murphy et al., 2008). This led us to assess the status of tyrosine-hydroxylase-positive dopaminergic neurons in wild-type and *CG2135*^−/−^ fly brains. A significant loss of dopaminergic neurons was seen in 30-day-old *CG2135*^−/−^ fly brain as compared to the age-matched control (Fig. 4Q-S). This loss might be responsible, at least in part, for the locomotion defect seen in the *CG2135*^−/−^ flies. Furthermore, analysis of the retinal architecture revealed a gross defect in ommatidial organization (appearance of holes between the ommatidia) and occasional loss of photoreceptors in the 30-day-old *CG2135*^−/−^ fly. Such signs of retinal degeneration were barely detectable in the age-matched wild-type fly, in which the overall ommatidial architecture and the photoreceptors were well preserved (Fig. 4T,U). The ocular phenotype in the *CG2135*^−/−^ fly reported here, which mimics the photoreceptor degeneration earlier seen in the MPS VII mouse, may explain why visual impairment is a common occurrence in MPS VII patients (Lazarus et al., 1993; Montaño et al., 2016). As expected, the neuropathological abnormalities in the *CG2135*^−/−^ fly were reverted to a significant extent by transgenic overexpression of the *CG2135* gene in the homozygous knockout background (Fig. S7).

### Muscle degeneration in the *CG2135*^−/−^ fly

**Fig. 5.**
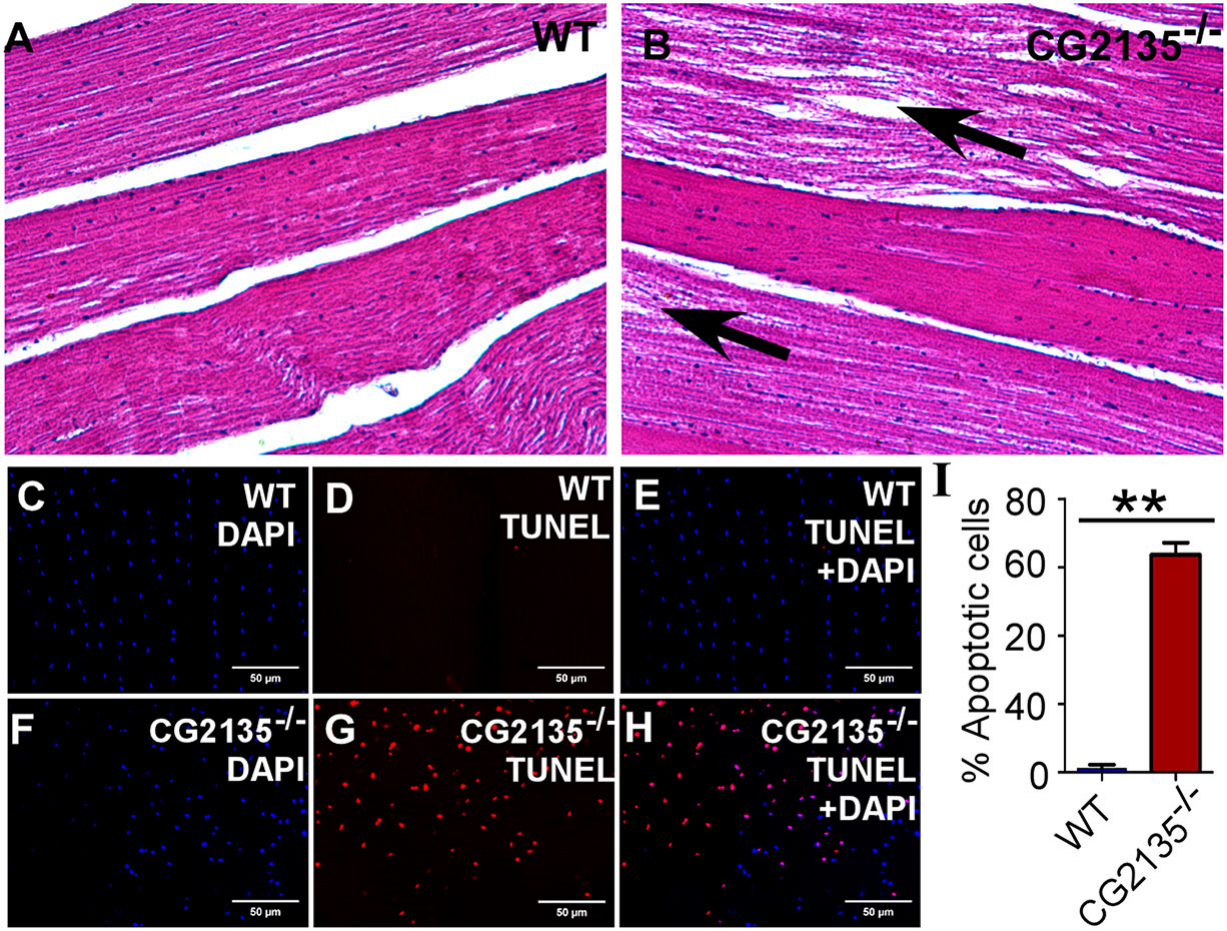
**Muscle degeneration in the *CG2135*^−/−^ fly.** (A,B) Hematoxylin and eosin staining of longitudinal sections of thoracic muscle of 30-day-old flies, showing intact muscle structure in the wild type (WT) fly as opposed to fragmented muscle fibres (indicated by arrows) in the *CG2135*^−/−^ fly. (C-H) TUNEL staining (red) of the thoracic muscles, showing widespread apoptosis in the 30-day-old *CG2135*^−/−^ fly. The total number of cells is represented by DAPI (blue). (I) Bar graph showing that the percentage of TUNEL-positive (apoptotic) cells in the muscle of the *CG2135*^−/−^ fly is significantly higher than in the WT fly. Error bars represent the s.e.m. of values from four independent experiments. **P≤0.01.

Apart from neurodegeneration, a defect in muscle could be another pathological basis for locomotor disability in *CG2135*^−/−^ flies. Indeed, histological analysis of the thoracic muscles revealed significant loss of muscle fibre integrity in the 30-day-old *CG2135*^−/−^ flies but not in the age-matched control flies or in the transgenic rescue fly (Fig. 5A,B, Fig. S8). TUNEL staining showed extensive apoptosis in muscles of the *CG2135*^−/−^ flies (Fig. 5C-H). The percentage of the apoptotic nuclei in muscles of the *CG2135*^−/−^ flies was >60% as compared to only ∼1% in the wild-type flies (Fig. 5I). These data indicate that muscle degeneration in the *CG2135*^−/−^ flies is due to extensive apoptosis of the myocytes, which may contribute to impaired locomotion in these flies.

### Resveratrol attenuated neuromuscular degeneration and locomotor disability in the *CG2135*^−/−^ fly

**Fig. 6.**
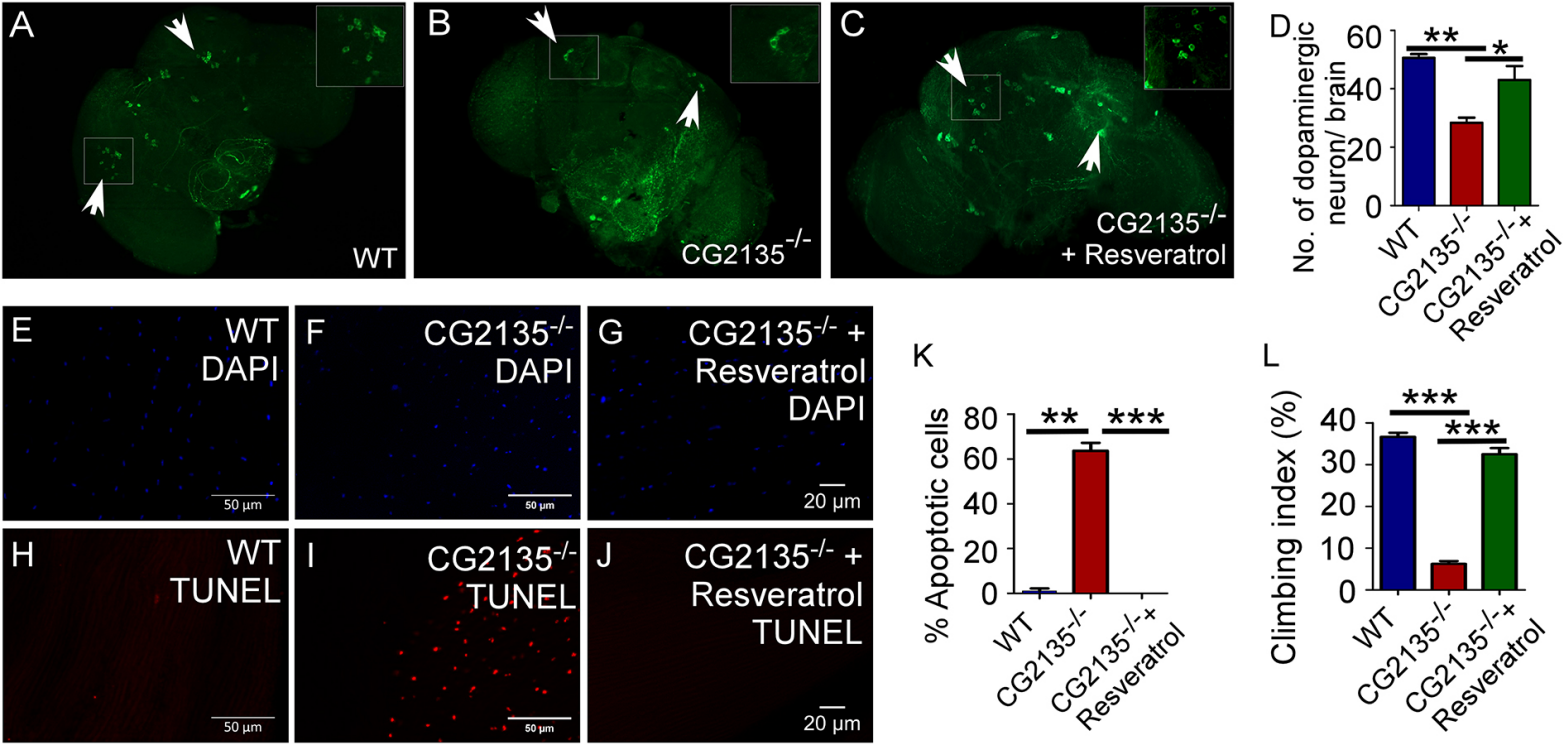
**Attenuation** **of** **neuromuscular degeneration and locomotor deficits in the** ***CG2135*^−/−^ fly by resveratrol treatment.** (A-C) Immunostaining of the 30-day-old fly brains with anti-tyrosine-hydroxylase antibody (green), showing the number of dopaminergic neurons in 30-day-old WT and *CG2135*^−/−^ flies fed on normal diet as opposed to the age-matched *CG2135*^−/−^ fly fed with resveratrol. Enlarged view of a dopaminergic neuron cell body from the boxed regions of the brain is provided in the insets. (D) Bar graph represents the number of dopaminergic neuron cell bodies in the fly brains. (E-J) TUNEL staining (red) of the thoracic muscles, showing widespread myocyte apoptosis in the *CG2135*^−/−^ fly but not in the WT fly or resveratrol-fed *CG2135*^−/−^ fly. The total number of nuclei is marked by DAPI staining (blue). (K) Percentage of TUNEL-positive nuclei (apoptotic cells) in the 30-day-old WT and the *CG2135*^−/−^ fly fed on normal diet as opposed to the age-matched *CG2135*^−/−^ fly fed with resveratrol. (L) Climbing index of 30-day-old WT and the *CG2135*^−/−^ flies fed on normal diet as opposed to the age-matched *CG2135*^−/−^ fly fed with resveratrol (*N*∼200). Error bar represents s.e.m. of values from >three independent experiments. *P≤0.05, **P≤0.01, ***P≤0.001.

Resveratrol, a natural polyphenol, has been shown to exert multiple health benefits in a variety of animal models (Baur and Sinclair, 2006). This includes, lifespan extension, delaying the age-related phenotypes and prevention of diseases such as cancer or diabetes (Howitz et al., 2003; Jang et al., 1997; Lagouge et al., 2006; Valenzano et al., 2006; Wood et al., 2004). The neuroprotective role of resveratrol is also well documented (Bastianetto et al., 2015; Kim et al., 2007; Parker et al., 2005). These promising results prompted us to test whether resveratrol can attenuate neuromuscular degeneration and associated locomotor disability seen in the older *CG2135*^−/−^ flies. We observed that the 30-day-old *CG2135*^−/−^ flies, when fed with resveratrol, had significantly more dopaminergic neurons in their brain as compared to those maintained on a standard diet. The number of dopaminergic neurons in the resveratrol-fed *CG2135*^−/−^ flies was almost similar to that observed in the wild type flies (Fig. 6A-D). The 30-day-old *CG2135*^−/−^ flies on the resveratrol diet did not exhibit any signs of apoptosis in the muscles. This is in stark contrast with extensive muscle cell apoptosis seen in the age-matched *CG2135*^−/−^ flies reared on standard diet (Fig. 6E-K). Furthermore, suppression of neuromuscular degeneration in the 30-day-old resveratrol-fed *CG2135*^−/−^ flies was accompanied by near-complete recovery of their climbing ability (Fig. 6L). This suggests that therapeutic administration of resveratrol can be explored as an alternative approach to management of MPS VII.

## DISCUSSION

We report here the generation and characterization of a novel *β-GUS* knockout (*CG2135*^−/−^) fly that closely resembles the MPS VII disease phenotypes. The cardinal features of MPS VII, such as reduced lifespan, progressive decline in locomotor activity and vacuolation, as well as accumulation of storage materials in the brain, were manifested in this fly. The *CG2135*^−/−^ fly thus represents the first invertebrate model of MPS VII and has proven to be extremely useful for unravelling mechanistic complexities of the disease and exploring therapeutic possibilities.

The locomotor deficit in the *CG2135*^−/−^ fly was quite striking. This could be attributed to neuromuscular degeneration, particularly the loss of dopaminergic neurons and muscle cell apoptosis. The adult fly brain contains eight different clusters of dopaminergic neurons that are involved in different functions (Mao and Davis, 2009). Using cluster-specific drivers of α-synuclein expression, the protocerebral anterior medial (PAM) cluster dopaminergic neurons were shown to be important for locomotor control in a *Drosophila* model of Parkinson's disease (Riemensperger et al., 2013). Hence, it is likely that selective degeneration of PAM-cluster dopaminergic neurons underlies the locomotor defect of the *CG2135*^−/−^ fly. Apoptotic muscle degeneration seen in this fly may further worsen their motor function. A link between muscle cell apoptosis and movement disability was earlier established in a *Drosophila* model of autosomal recessive juvenile parkinsonism by knocking out the *parkin* gene, which encodes a mitochondrial ubiquitin-protein ligase (Greene et al., 2003). Although the status of dopaminergic neurons or muscle pathology is yet to be studied in MPS VII patients or in the mouse model, our findings suggest that, in addition to skeletal abnormalities, dopaminergic neuronal loss and muscle wasting are major contributing factors for restricted mobility seen in the majority of these patients (Montaño et al., 2016). Further characterization of the *CG2135*^−/−^ fly may reveal additional phenotypes, including some of the complex neurological and metabolic aspects of this disease.

One major advantage with this *Drosophila* model of MPS VII is that it can be used for rapid screening of small molecules to identify new therapeutic leads (Fernández-Hernández et al., 2016; Giacomotto and Ségalat, 2010). As a proof of this concept, we tested the efficacy of resveratrol, which has emerged as a promising compound having potential health benefits (Bastianetto et al., 2015; Baur and Sinclair, 2006; Howitz et al., 2003; Jang et al., 1997; Kim et al., 2007; Lagouge et al., 2006; Parker et al., 2005; Valenzano et al., 2006; Wood et al., 2004). Compelling evidence was presented for resveratrol-mediated protection against neuromuscular degeneration and progressive locomotor disability in the *CG2135*^−/−^ fly. These results are consistent with previous reports where resveratrol treatment was shown to protect against chemically induced depletion of dopaminergic neurons or muscle wasting (Hori et al., 2011; Okawara et al., 2007; Zhang et al., 2010). However, the mechanism by which resveratrol could rescue the *CG2135*^−/−^ fly is yet unclear. Antioxidant, anti-inflammatory and autophagy-modulating properties of resveratrol are widely reported (Bastianetto et al., 2015; Hasima and Ozpolat, 2014; Hori et al., 2011; Zhang et al., 2010). Also, resveratrol-mediated activation of AMPK has been proposed to control cellular energy homeostasis and confer neuroprotection (Dasgupta and Milbrandt, 2007). These plausible mechanistic pathways remain to be explored in our model to establish the mode of action of resveratrol. Nevertheless, our results provide a strong impetus for further studies to explore the possible use of resveratrol as an alternative treatment strategy for MPS VII.

Apart from being a convenient drug screening platform, the *CG2135*^−/−^ fly may also be utilized for large-scale genetic screenings leading to identification of modifiers of the disease phenotype (St Johnston, 2002). Such carefully designed screens have earlier led to the identification of genetic suppressors of polyglutamine toxicity in flies (Kazemi-Esfarjani and Benzer, 2000). Therefore, this novel model of MPS VII, combined with the power of fly genetics, holds the key to deeper exploration of the disease mechanism and drug discovery. The findings derived from this model may also have broader implications in understanding and managing other closely related MPS disorders.

## MATERIALS AND METHODS

Unless specified otherwise, all reagents were from Sigma-Aldrich. Details of all the primers (IDT) are provided in Table S1.

### *Drosophila* strains and maintenance

*w*^1118^, actin 5C Gal4, FLP, I-*Sce*I and balancer flies were all obtained from the Bloomington Drosophila Stock Center at Indiana University, USA. Flies were maintained at 25°C, at controlled density, with 12 h day-night cycle on a standard cornmeal agar medium. Wherever indicated, 400 µM resveratrol (Calbiochem) was added in the fly food.

### Cell culture

S2 cells (kindly provided by Dr Sankar Maiti, IISER Kolkata, India) were cultured at 28°C in Schneider's medium supplemented with 10% fetal bovine serum (FBS; Gibco), 2 mM L-glutamine, 100 U/ml penicillin and 100 μg/ml streptomycin.

### β-GUS assay

As described previously, β-GUS activity was measured fluorometrically using 4-methylumbelliferyl β-D-glucuronide as substrate (Glaser and Sly, 1973). Briefly, tissues/cells were lysed by homogenization/sonication and the assay was performed at 37°C for varying times using 100 µl of the substrate solution containing 10 mM 4-methylumbelliferyl β-D-glucuronide and 1 mg/ml BSA in 0.1 M acetate buffer, pH 4.8. The reaction was stopped by adding glycine-carbonate buffer, pH 10.5, following which the product was quantified fluorometrically with respect to a standard curve generated using known concentrations of 4-methylumbelliferone. The β-GUS activity was normalized to protein concentration, determined by Lowry's method (Lowry et al., 1951).

### RNA isolation and RT-PCR

Total RNA from tissues was isolated using TRIzol reagent (Thermo Fisher Scientific). DNA contaminants were removed by DNase I (Invitrogen) treatment. DNA-free RNA (1 µg) was reverse transcribed using a cDNA synthesis kit (Invitrogen). The cDNA was used as a template for the PCR reactions with specific primers.

### Cloning, expression and protein purification

Coding regions of the *CG2135* and *CG15117* genes were PCR amplified from the whole fly cDNA using gene-specific primer sets P7/P8 and P9/P10, and cloned in the pMT-puro vector (Addgene). Designing of the reverse primers ensured in-frame addition of a hexa-histidine coding sequence at the C-terminal end of the cDNAs. The clones were verified by sequencing, following which the constructs were transfected in S2 cells by calcium phosphate method (Sambrook and Russell, 2016). Stably transfected S2 cells were selected in puromycin-containing medium. For protein induction, the cells were grown for 96 h in serum-free medium in the presence of 500 µM CuSO_4_. Since a substantial amount of the overexpressed protein was secreted out of the cell (as indicated by β-GUS assay), a total of 300 ml of the conditioned media was collected in batches and used for protein purification. The media was subjected to ammonium sulfate precipitation in three stages: 0-30%, 30-65% and 65-80%. For both CG2135 and CG15117, maximum β-GUS activity was detected in the 30-65% pellet fraction, which was dissolved in a minimum volume of 50 mM sodium phosphate buffer (pH 8.0) containing 10 mM imidazole and 300 mM sodium chloride. The solutions were extensively dialyzed against the same buffer and the recombinant enzymes were purified to homogeneity by Ni-NTA (Qiagen) affinity chromatography, following the manufacturer's suggested protocol. Protein purity was confirmed by SDS-PAGE followed by silver staining.

### Generation of *CG2135* knockout and rescue flies

The *CG2135*^−/−^ fly was generated by ends-out gene targeting (Rong and Golic, 2000). For this, 5′ and 3′ untranslated regions (UTRs) of the *CG2135* gene (corresponding to the 3R:31771758-31774848 and the 3R:31768557–31771547genomic regions of the *Drosophila melanogaster* genome, respectively) was PCR amplified using P11/P12 and P13/P14 primer sets. The amplified 5′ and 3′ UTRs were respectively subcloned into *Not*1 and *Kpn*I/*Bam*HI restriction sites of the pw35Gal4 vector (a kind gift from Dr Craig Montell, USCB, USA). The targeting construct was verified by sequencing and then used to generate a donor transgenic fly (in *w*^1118^ background) by germline transformation (embryo microinjection service provided by C-CAMP, Bangalore, India). For targeting of the *CG2135* gene, the donor transgenic fly was crossed with the *w*[1118];P{ry[+t7.2]=70FLP}23 P{v[+t1.8]=70I-SceI}4A/TM3,Sb[1] fly (Bloomington stock number 6935). The larval progenies were heat-shocked at 37°C for 1 h for two subsequent days to induce FLP and I-*Sce*I expression. The targeting construct was excised from the genome as a circular DNA by FLP recombinase, which was subsequently linearized by I-*Sce*I endonuclease. The highly recombinogenic linearized targeting construct can, in principle, disrupt the *CG2135* gene in somatic as well as germ cells by replacing a 212-bp coding sequence with the targeting construct containing mini-*white* and *Gal4* markers. Flies in which such homologous recombination has happened were selected based on a red-white mosaic eye pattern (Maggert et al., 2008). Those flies were individually mated to *w*^1118^; Pin/Cyo;TM2, Ubx[130] e[s]/TM6B,e[1] Tb[1] balancer fly. Red-eyed progenies were selected and backcrossed to the same balancer fly to obtain the *CG2135*^−/−^ fly. Balancer and marker from the second chromosome were replaced with wild chromosome by using *w*^1118^; +/+; TM2, Ubx[130] e[s]/TM6B,e[1] Tb[1] to get pure *w*^1118^; +/+; *CG2135*^−/−^ flies. For generation of the rescue fly, *CG2135* cDNA was PCR amplified using the P7/P21 primer set and subcloned into the *Xho*I site of the pUAST vector (DGRC). The clone was verified by sequencing. The construct was microinjected in *w*^1118^ embryos and progeny was selected on the basis of the red eye colour marker. The fly containing the construct in the second chromosome was selected by chromosomal mapping. The actin 5C *Gal4* driver was used to drive the *UAS*-*CG2135* in rescue flies. Genotypes of the knockout and rescue flies were confirmed by genomic PCR with appropriate primer sets.

### Lifespan analysis

Total 120 newly eclosed flies were used for lifespan analysis. Males and females were separated and 20 flies were placed per vial. The flies were transferred to fresh vials every alternate day and the number of dead flies was recorded. Mean lifespan was calculated from the survivorship curve that represents percent survival with increasing age. Maximum lifespan was calculated as average age of the top 10% long-lived flies (Lushchak et al., 2012).

### Climbing assay

The climbing assay was performed as described earlier (Strauss and Heisenberg, 1993; Venkatachalam et al., 2008). For each set, 20-25 flies were acclimatized for 24 h and then transferred to a 50 ml graduated cylinder. The flies were gently tapped to the bottom, following which they immediately tend to climb up because of their negative geotactic behaviour. The climbing index was calculated as the percentage of flies that could climb up to the 25 ml mark within 15 s. Each experiment was performed several times with more than 100 flies.

### Egg laying and hatching assays

Freshly eclosed healthy male and female flies were kept separated for 3 days to attain maturity, following which they were mated at a 1:1 male:female ratio. As soon as the first batch of embryos emerged, the female flies were placed in separate wells with fly food. After 24 h, the flies were discarded and the number of embryos laid per day was counted. Hatching assay was performed in a 35 mm dish with fly food, each containing 50 freshly collected embryos. A moist paper was kept at the side of each plate to prevent drying. The number of unhatched embryos was counted after 48 h.

### Tissue processing, staining and imaging

For brain histology, the tissues were fixed in 2.5% glutaraldehyde for 4 h followed by post-fixation in 2% osmium tetraoxide for 2 h. The tissues were then subjected to ethanol dehydration and embedded in Epon. Semi-thin transverse sections (0.5 µm) were prepared and were stained with 0.1% Toluidine Blue. For examination of the retinas, whole fly heads were similarly fixed with glutaraldehyde–osmium-tetroxide and embedded in Epon. Tangential retinal sections (0.5 µm) were stained with Toluidine Blue. The thoracic muscles were fixed with 4% paraformaldehyde overnight. The fixed tissues were dehydrated in increasing concentrations of ethanol, infiltrated with paraffin wax, followed by embedding in paraffin moulds. Finally, thick sections (4 µm) were prepared and stained with haematoxylin and eosin. All tissue sections were analyzed by light microscopy. For whole-mount immunohistochemistry, the fly brains were dissected in S2 media supplemented with 15% FBS. Immediately after dissection, the tissues were fixed in 4% paraformaldehyde for 30 min and immunostaining was performed as described previously (Wu and Luo, 2006). Briefly, the tissues were incubated overnight with primary antibody at 4°C followed by washing three times. The following primary antibodies were used at the indicated dilutions: rabbit anti-tyrosine hydroxylase (Merck Millipore; 1:400) or rabbit anti-ubiquitin (Cell Signaling; 1:200). Anti-rabbit Alexa-Fluor-488 or anti-mouse Alexa-Fluor-568 goat secondary antibodies (Molecular Probes; 1:500, 3 h incubation at room temperature) were used for detection. LysoTracker Red (100 nM) and MitoTracker Red (250 nM) staining was done in live brain tissues following the manufacturer's (both from Thermo Fisher Scientific) instructions. After staining, the tissues were mounted in Vectashield (Vector Laboratories) mounting medium and fluorescence imaging was performed with Apotome.2 microscope (Carl Zeiss). A total of 9-12 image tiles were taken using the motorized stage where images of 5 slices of 0.32 μm were present in each tile. The images were reconstructed by the microscope's own software (ZEN) and analyzed. Mean fluorescence intensity in the *Drosophila* whole-brain image was calculated using ImageJ software. TUNEL staining of muscle sections was performed as per the manufacturer's (Roche) instructions.

### Statistical analysis

Statistical analyses were performed by paired two-tailed Student's *t*-test using GraphPad Prism. The results were expressed as the mean±s.e.m. from at least three independent experiments. *P*-values ≤0.05 were considered as statistically significant as indicated by asterisks: *P≤0.05, **P≤0.01 and ***P≤0.001.

## Supplementary Material

Supplementary information
