# Supplementary material for: Neuromuscular degeneration and locomotor deficit in a Drosophila model of mucopolysaccharidosis VII is attenuated by treatment with resveratrol
Source: Dis Model Mech. 2018 Nov 20;11(11):dmm036954. doi: 10.1242/dmm.036954 (PMC6262814; doi:10.1242/dmm.036954)
Supplement: Supplementary information [file dmm-11-036954-s1.pdf]

Table S1: Primers used in this work

Primer	Primer sequences (5' → 3')	Purpose
P1	GCACTCACTTCCCGCCTTT	Semi Quantitative RT PCR of CG2135
P2	GTAATAGTCTGCGCGGTCCG	
P3	GGCTTGCATATGCAACCTGC	Semi Quantitative RT PCR of CG15117
P4	CGGGTGTAGCTTTGTGCTGTC	
P5	ACAACGGCTCTGGCATGTG	Semi Quantitative RT PCR of Actin 5C
P6	GGGACGTCCCACAATCGATG	
P7	GCGCCTCGAGATGAAAAGTCATTGGATGCCCGTCACC	Cloning of CG2135 cDNA in pMT puro and pUAST vector
P8	GCGCCTCGAGAAGCTCGTTATGGATATGCTTGGAAATGTAAAC	Cloning of CG2135 cDNA in pMT puro vector
P9	GCGCCTCGAGATGGCTCTGTCCATCTTCTCCCTG	cDNA of CG15117 clone in pMT puro vector
P10	GCGCCTCGAGGGATATCAGATCGGCGATGTAGGTGAAG	
P11	GCGCGGTACCCTGAAATCAGAATATTTCTATCGAGTTAC	Clone of extended 5' flanking region of CG2135 in PW35GAL4 vector
P12	GCGC GGATCC AAA GGA AAT CAA TAC CAG CCA	
P13	CAGAGCGGCCGCATCTGTGACCGGTCTGAGTC	Clone of extended 3' flanking region of CG2135 in PW35GAL4 vector
P14	CAGAGCGGCCGCTGGAATGTGGCAACTGGTTAG	
P15	GGACAAGGTGCAGCAGATGAAG	Knockout confirmation (knockout construct inserted in right place)
P16	TCATCCTCTTCAGACCAATCAAATCCATG	
P17	GTTCACTATATTTGCGCTGGTCACACT	Knockout confirmation (confirmation of deletion)
P18	GTGACGGGCATCCAATGACTTTTC	
P19	ATGAAAAGTCATTGGATGCCCGTCACC	Knockout confirmation (absence of full length CG2135 gene)
P20	TCAAAGCTCGTTATGGATATGCTTGGAAATGTAAAC	
P21	GCGCCTCGAGTCAAAGCTCGTTATGGATATGCTTGGAAATGTAAA C	Cloning of CG2135 cDNA in pUAST vector

The restriction sites of the primers are underlined.

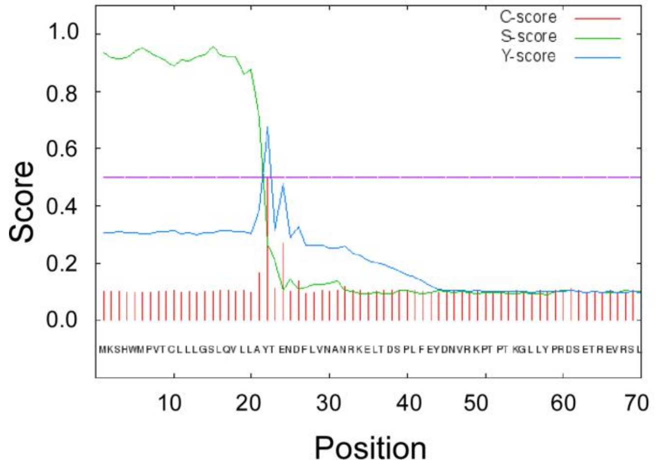

Fig. S1. Prediction of ER signal sequence in CG2135. SignalP 4.1 prediction showing a cleavable 21 amino acids present at N terminal (green line) with cleavage site between 21st and 22nd amino acids (red line).

Disease Models & Mechanisms • Supplementary information

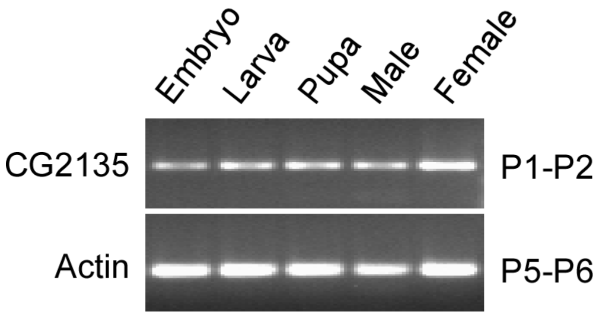

Fig. S2. Expression profile of the CG2135 mRNA. Agarose gel image of CG2135 RT PCR products (amplified with primers P1 and P2) confirming expression of the CG2135 mRNA in all developmental stages of *Drosophila*. Actin was amplified (with primers P5 and P6) as internal control.

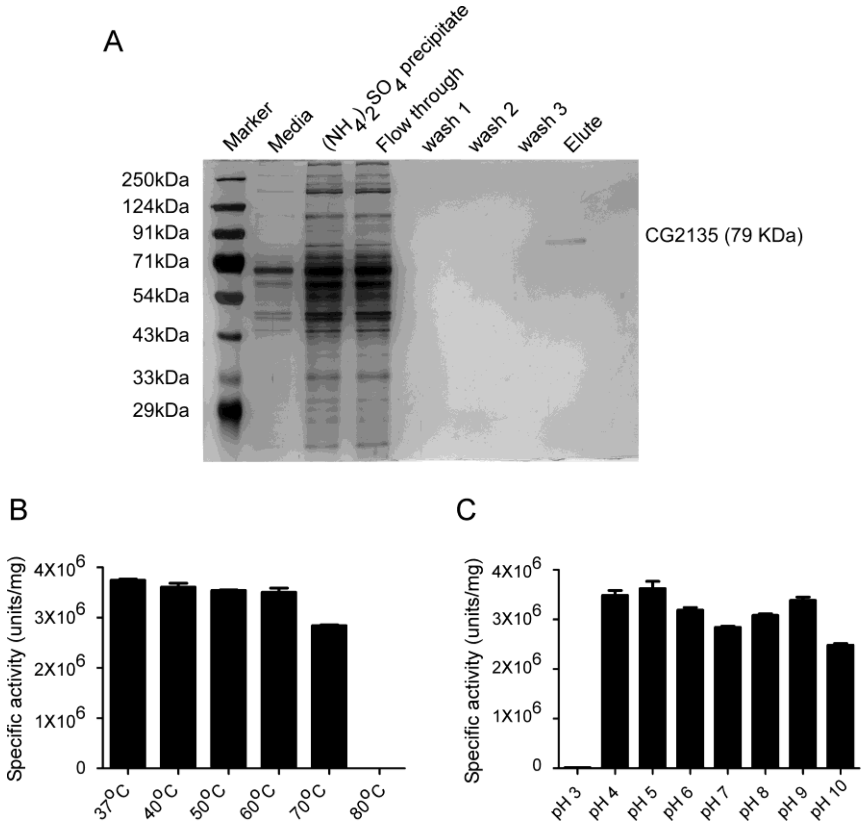

Fig. S3. Purification and biochemical characterization of CG2135. (A) CG2135 protein was purified from stably expressing S2 cell culture medium. Different fractions (as indicated in the figure) was subjected to SDS-PAGE and the gel was silver stained to visualize the proteins. Purity of the CG2135 protein is indicated by a single band at the correct molecular weight (75 kDa). (B) Bar graph represents specific activity of CG2135 after 30 min incubation at different temperature as indicated in the figure. (C) Bar graph represents β-GUS activity of CG2135 at different pH range from pH 3 to 10. Error bar represents SEM of values from 3 independent experiments.

Disease Models & Mechanisms • Supplementary information

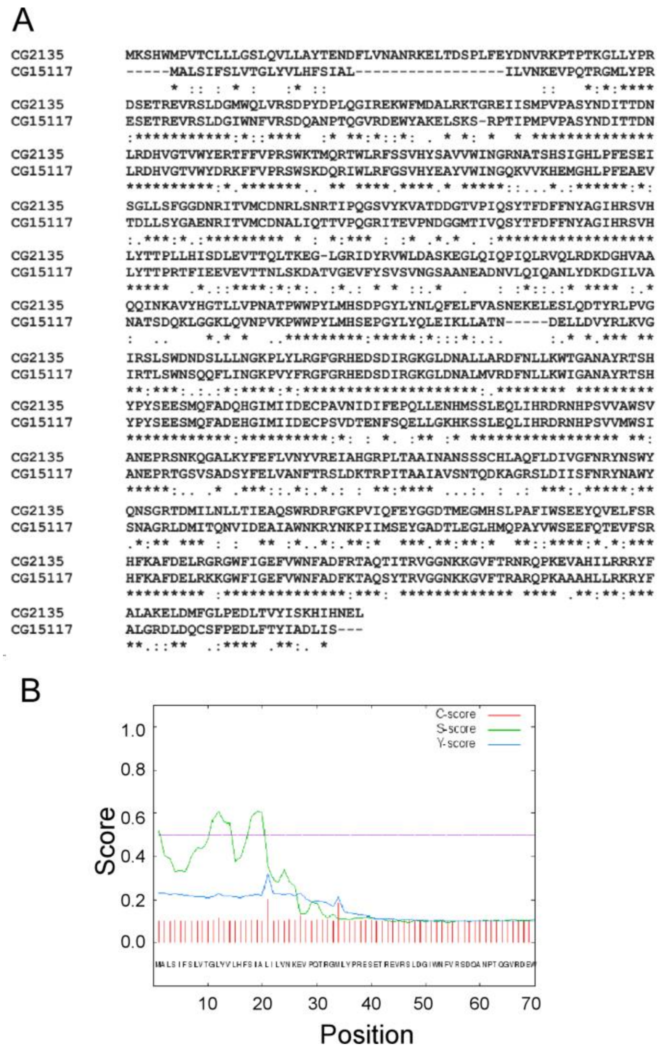

Fig. S4. Sequence analysis of CG15117. (A) Amino acid sequence alignment of CG15117 with that of CG2135, revealing more than 60% identity between these two protein sequences. (B) Analysis of CG15117 protein sequence with SignalP 4.1 prediction server, showing absence of any cleavable N-terminal signal sequence.

Disease Models & Mechanisms • Supplementary information

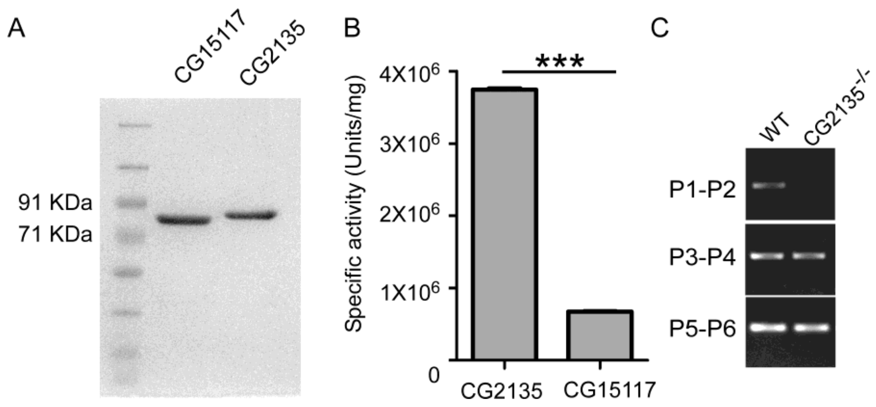

Fig. S5. Characterization of CG15117 activity and expression. (A) Silver stained protein gel showing purified CG15117 and CG2135 as single bands at their predicted molecular weights 75 and 79 kDa, respectively. (B) Bar graph representing β-GUS specific activities of CG2135 and CG15117 showing CG2135 is 6 times more active than CG15117; error bar representing SEM of values from 3 independent experiments. (C) RT-PCR with indicated primer sets showing CG2135 mRNA is absent in $CG2135^{-/-}$ fly (first row) but CG15117 is expressed in the WT as well as in the $CG2135^{-/-}$ fly (middle row). Actin 5c was used as internal control (bottom row).

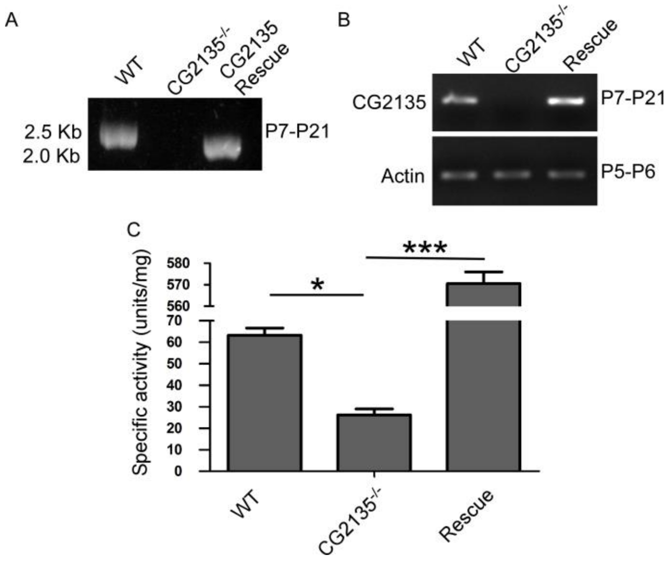

Fig. S6. Confirmation of the CG2135 rescue fly. (A) Genomic PCR using full length CG2135 primers showing amplification of a 2.5 Kb genomic fragment (containing introns) in the WT fly and a 2 Kb fragment corresponding to the CG2135 cDNA (intronless) in the CG2135$^{-/-}$ fly. (B) RT-PCR with CG2135 specific primer sets (P7-P21), showing complete absence of the CG2135 mRNA in the CG2135$^{-/-}$ fly as opposed to the WT fly and the CG2135 rescue fly. RT-PCR with Actin 5C primers (P5-P6) was used as internal control. (C) Bar graph representing β-GUS specific activity of WT, CG2135$^{-/-}$ and CG2135 transgenic rescue fly. Error bar representing SEM of values from 3 independent experiments. Significantly higher β-GUS activity in the rescue fly confirms transgenic overexpression of the gene in these flies.

Disease Models & Mechanisms • Supplementary information

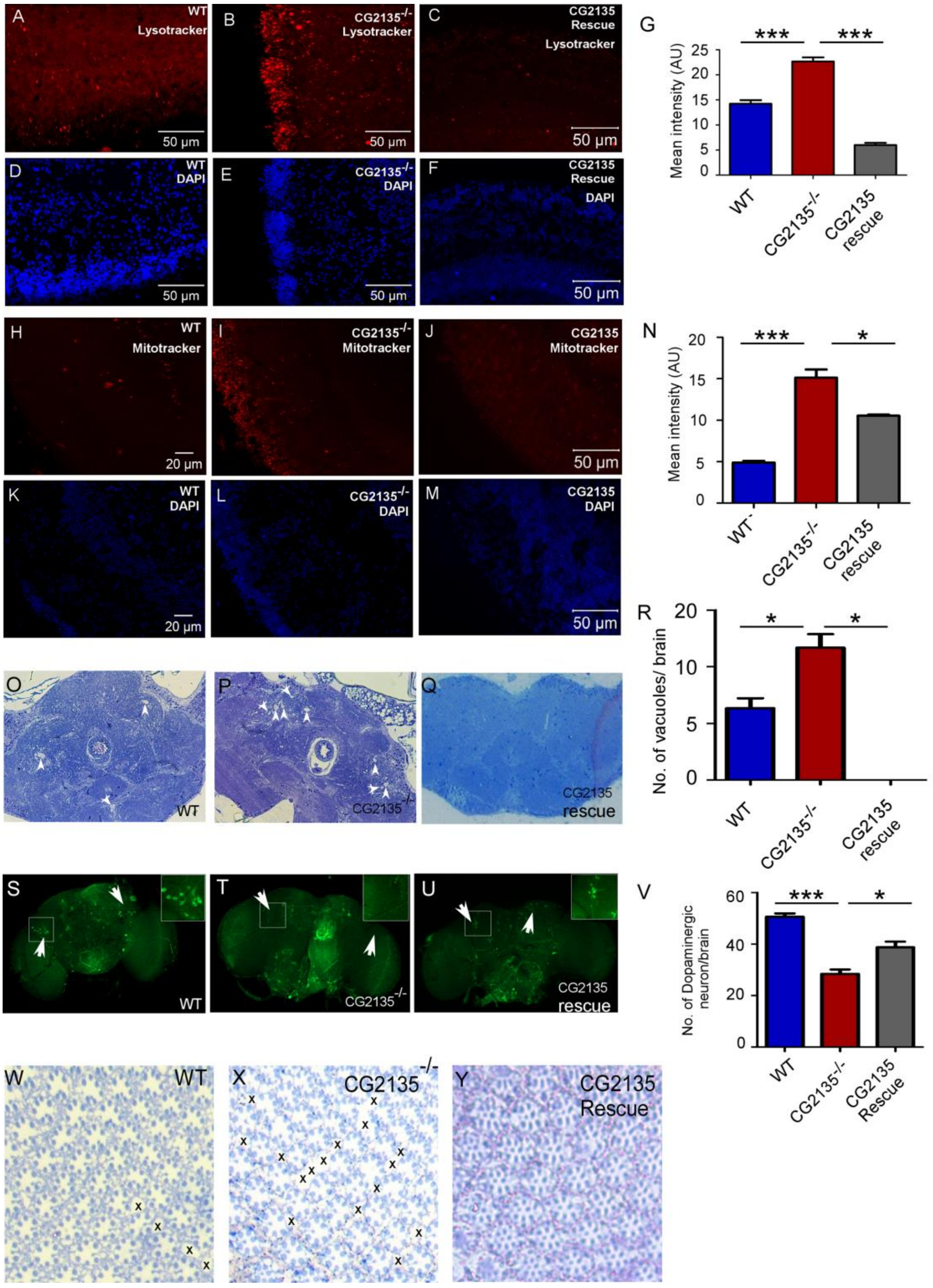

Fig. S7. Rescue of neuropathological abnormalities in the CG2135$^{-/-}$ fly by transgenic overexpression of CG2135. (A-F) Lysotracker (red) and DAPI (blue) staining of the 30-day old fly brains showing increased abundance of the lysotracker positive vesicles in the CG2135$^{-/-}$ fly compared to the wild type (WT), which was reverted to a significant extent in the CG2135 rescue fly. (G) Bar graph showing mean intensities of lysotracker staining in the

respective fly brain. (H-M) MitoTracker (red) and DAPI (blue) staining of the 30-day old fly brains showing increased mitochondrial accumulation in the CG2135$^{-/-}$ fly brain compared to the wild type (WT), which was reduced to a significant extent in the CG2135 rescue fly. (N) Bar graph showing mean intensities of mitotracker staining in the respective fly brain. (O-Q) Toluidine blue stained brain sections of the 30-day old flies showing increased vacuolation (indicated by arrows) in the CG2135$^{-/-}$ fly compared to the wild type (WT), which was almost non-existent in the CG2135 rescue fly. (R) Quantification of the number of vacuoles in the fly brain sections. (S&U) Immunostaining of the 30-day old fly brains with anti-tyrosine hydroxylase antibody (green) showing loss of dopaminergic neurons in CG2135$^{-/-}$ fly brain compared to the WT flies, which was reverted to some extent in the CG2135 rescue fly. Enlarged view of dopaminergic neuron cell body from the boxed regions of the brain is provided in the index. (V) Quantitation of dopaminergic neurons in the respective fly brains. (W-Y) Toluidine blue stained retinal sections showing well-preserved ommatidial architectures in the 30-day old WT fly as opposed to defective ommatidial organization (cross marks showing the gaps) in the age matched CG2135$^{-/-}$ fly. The ommatidial architectures in the 30-day old CG2135 rescue fly is almost normalised.

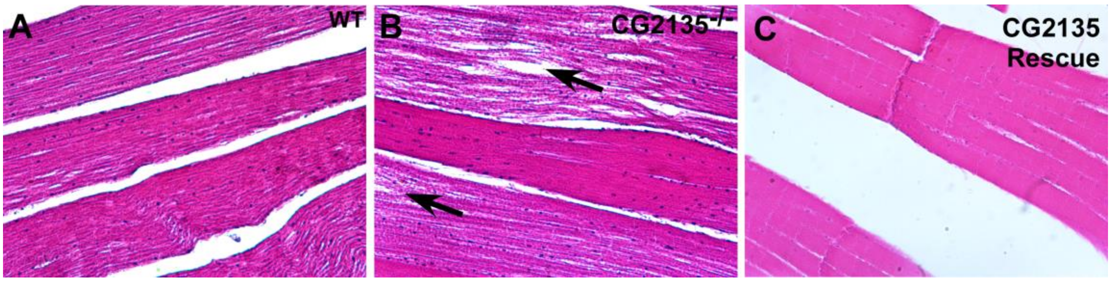

Fig. S8. Rescue of muscle degeneration in the CG2135$^{-/-}$ fly by transgenic overexpression of CG2135. (A-C) Hematoxylin and eosin staining of longitudinal section of thoracic muscle of 30-day old flies showing intact muscle structure in the wild type (WT) fly and the CG2135 rescue fly as opposed to fragmented muscle fibres (indicated by arrows) in the CG2135$^{-/-}$ fly.
